# Characterizing Micromechanical Properties of Friction Welding Interface between TiAl Alloy and GH3039 Superalloy

**DOI:** 10.3390/ma13092072

**Published:** 2020-04-30

**Authors:** Suigeng Du, Songlin Wang, Wanting Xu

**Affiliations:** Key Laboratory of High Performance Manufacturing for Aero Engine, School of Mechanical Engineering, Northwestern Polytechnical University, Xi’an 710072, China; fwcenter@nwpu.edu.cn (S.D.); xuwanting@mail.nwpu.edu.cn (W.X.)

**Keywords:** friction welding, micromechanical properties, nanoindentation, fracture toughness, in-situ tensile, TiAl alloy

## Abstract

To explore the macro-fracture mechanism of a friction welded joint between TiAl alloy and GH3039 superalloy, the micromechanical properties of intermediate phases at the joint interface are characterized and the relationship between the macro-fracture and micro-fracture of the joint is established. The indentation technique has been employed to calculate the nano-hardness and fracture toughness of the intermediate phases. The dynamic in-situ tensile test in SEM has been applied to observe the initiation and propagation process of cracks at the interface. It has been found that Al_3_NiTi_2_ and AlNi_2_Ti have the highest nano-hardness and elastic recovery rates, while TiAl and GH3039 base metals have the lowest nano-hardness and elastic recovery rates. This indicates that the harder the materials, the more prone they are to elastic deformation. Nevertheless, the fracture toughness of Al_3_NiTi_2_ and AlNi_2_Ti are the two lowest, which were 1.7 MPa·m^1/2^ and 2.7 MPa·m^1/2^, respectively. The cracks sprouted from Al_3_NiTi_2_ and AlNi_2_Ti and then spread throughout the entire intermediate phase zone. In other words, the fracture mainly happened in these two phase layers. It has been concluded that Al_3_NiTi_2_ and AlNi_2_Ti were the two weakest phases at the interface and their poor fracture toughness results in low joint strength.

## 1. Introduction

TiAl-based alloys, as promising, high-temperature structural materials, have been widely used in aero-engine components due to their excellent features, such as low density, high specific strength, favorable oxidation resistance and high-temperature mechanical properties [1,2]. In particular, the turbine rotor of a turbocharger made by TiAl alloy can be lightened more than 50% compared with that made by a Ni-based casting superalloy [3]. Consequently, the transient response and power loss of the engine significantly increases and declines, respectively [4]. Therefore, research on the connection technology between TiAl turbines and steel shafts is particularly important [5,6]. GH3039 superalloy has been adopted as the interlayer to effectively implement the friction welding of TiAl to 42CrMo steel in a previous study [7]. To explore the macro-fracture mechanism and enhance the strength of the TiAl/GH3039 friction welded joint, it is necessary to characterize the micromechanical properties of the interface and establish the relationship between the macro-fracture and micro-fracture of the joint.

The researchers in [7] reveal that the TiAl/GH3039 joint interface is composed of five intermediate phases (Ti_3_Al, Al_3_NiTi_2_, AlNi_2_Ti, Ni_3_(Al,Ti), and (Ni,Cr)_ss_), but the axial width of each intermediate phase is narrow (about 1 to 3 μm). In the microhardness test, the indenter would inevitably cover a number of phases owing to its large indentation profile. Thus only comprehensive hardness of multiple phases can be measured, which does not characterize the hardness of a single intermediate phase. Meanwhile, nanoindentation has been acknowledged as a well-developed technique in characterizing the micro-nanohardness of the weld interface [8,9,10]. The nano-hardness of the thermo-mechanical affected zone (TMAZ), and stirred zone (SZ) of the AA6082 friction stir welding (FSW) interface were measured by Cabibbo et al. [11]. The nano-hardness of each phase at the Ti/Fe explosion welding interface (using Cu20V as filler) was obtained by Chu et al. [12].

Although the fracture toughness or the strength of phases cannot be directly characterized by nano-hardness, special phases with higher or lower hardness can be identified. The ability of materials to prevent crack propagation can be characterized by fracture toughness [13,14]. The indentation technique was applied to calculate the fracture toughness of single-phase materials or bulk intermediate phases at the weld interface [15,16]. Cai et al. [17] obtained the fracture toughness of WC–Fe cermet in W–WC–Fe composite, ranging from 4.1 to 7.9 MPa·m^1/2^. The result obtained by Casellas et al. [18] reveals that the fracture toughness of carbides in tool steels ranged from 2.2 to 3.7 MPa·m^1/2^. The fracture toughness of the bulk intermediate phase (Fe_2_Zr) at the Fe–Zr arc welding interface was characterized using nanoindentation by Chu et al. [19], and the average fracture toughness was 2.5 MPa·m^1/2^. However, the fracture toughness of the phases with narrow width has not been successfully characterized, to the authors’ knowledge.

The intermediate phases with poor fracture toughness would generally deteriorate the welded joints. The dynamic in-situ tensile test in SEM has a good performance in analyzing the deformation of brittle phases, the fracture mechanism of weld interface and the fracture position of welded joints [20,21,22]. Li et al. [23] identified the fracture position of TiAl/42CrMo brazed joint (with Ag–Cu–Ti as foil filler) and the Al–Cu–Ti ternary brittle phases which degraded the joint strength by the in-situ tensile test.

In this paper, the micromechanical properties of the friction welded joint between TiAl alloy and GH3039 superalloy were investigated. First, the nano-hardness of the phases was measured and special phases with higher hardness were found. Next, the weak phases with narrow width were identified by the fracture toughness. Finally, the micro-fracture mechanism of the joint was studied by an in-situ tensile test.

## 2. Experimental Procedures

### 2.1. Materials and Process

The TiAl alloy used in this study is a casting alloy made by the vacuum induction levitation melting technique. In order to reduce crystal defects and improve fatigue performances, the corresponding heat treatment was conducted by hot isostatic pressing (HIP) at 1250 °C under 120 MPa for 2 h and vacuum annealing at 950 °C for 2 h. The GH3039 alloy is a single phase (austenite) superalloy strengthening by the solid solution mechanism at 1080 °C. The chemical compositions of the base metals are given in Table 1. The diameter and length of the two samples were 25 mm and 60 mm, respectively. Prior to welding, the faying surfaces were polished by SiC paper and then cleaned by acetone.

**Figure 1:**
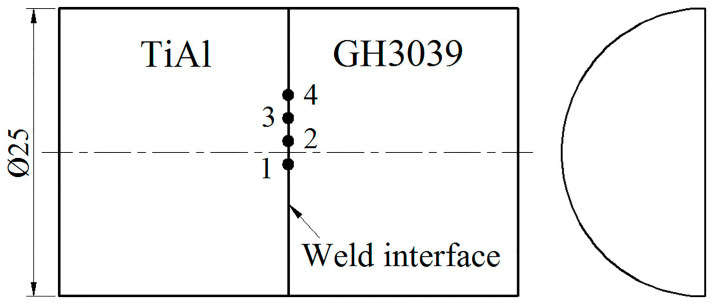
Location and orientation of the metallographic observation (mm): the distances of positions 1, 2, 3 and 4 from the axis are about −1 mm, 1 mm, 3 mm and 5 mm, respectively.

The friction welding was carried out using a continuous-drive friction welding machine (C500, 500 kN and 2000 rev/min machine capability) which was designed and manufactured by Northwestern Polytechnical University, Xi’an, China. The machine was equipped with a closed-loop control system. During welding, the friction force and upset force were maintained at 500 MPa and 560 MPa, respectively. The rotating speed was set to 1000 rev/min. The friction distance was 1.1 mm. After welding, the specimens were heat treated at 560 °C for 45 min and then cooled down to ambient temperature in air. The welded joints were cut in half along the axis and the metallographic specimens were made from a cross section of the joints through mechanical grinding and polishing. The interfacial microstructures were detected using a scanning electron microscope (SEM, Verios G4, FEI, Hillsboro, OR, USA). The location and orientation of the metallographic observation are described in Figure 1.

### 2.2. Characterization Methods

The nano-hardness and Young’s modulus (i.e., elastic modulus) of the base metals and intermediate phases at the interface were measured using a nanomechanical test instrumentation (TI980, Hysitron, Minneapolis, MN, USA, maximum load of 10 mN) equipped with a Berkovich diamond indenter. Load control tests were performed with peak loads of 3 mN at a constant loading rate of 0.6 mN/s. The indenter was held for a standard period of 2 s at each peak load. The nano-hardness and Young’s modulus of each phase were obtained at five measuring points.

**Figure 2:**
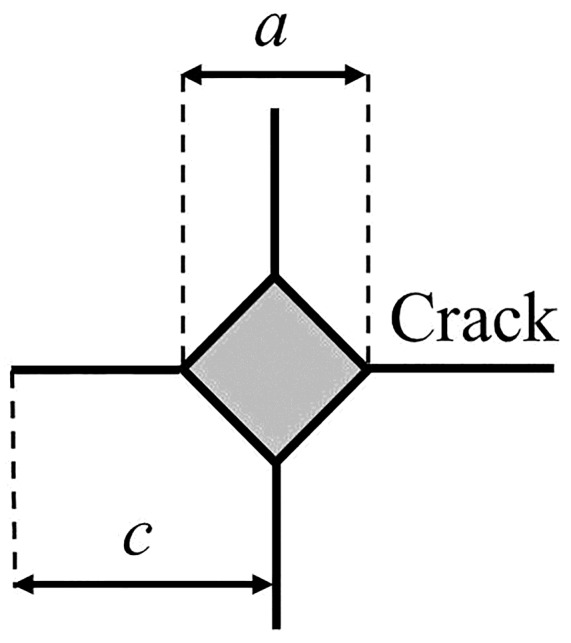
Radial cracks emanating from a Vickers indenter impression.

The fracture toughness of the base metals and intermediate phases was calculated by means of the indentation method using a microhardness tester (FM-800, Future-Tech, Kawasaki, Japan, maximum load of 10 N) equipped with a rectangular pyramid diamond indenter (angle of 136°). Load control tests were conducted with eight different peak loads *P* (0.1 N, 0.25 N, 0.5 N, 1 N, 2 N, 3 N, 5 N and 10 N). At each peak load, the indenter was held for a conventional period of 5 s. For a rectangular pyramid indenter with angle of 136 °, the fracture toughness can be calculated by [15,18]:

(1) $$\left\{ \begin{array}{l} H_{V}=1.8544\times \frac{P}{a^{2}}\times 10^{3}\\ K_{C}=\beta {\left(\frac{E}{H_{V}}\right)}^{1/2}\frac{P}{c^{3/2}}\times 10^{3} \end{array}\right.$$

where *K_C_* is the fracture toughness (MPa·m^1/2^), *E* is the Young’s modulus (GPa), *H_v_* is the Vickers hardness (GPa), *P* is the peak load (N), *a* is the diagonal length of indentation impression (μm) and *c* is the crack length (μm). The constant *β* is related to the indenter, which has been experimentally calibrated over a number of brittle materials and found to be 0.016 for the Vickers 4-sided pyramidal indenter [24]. The radial cracks emanating from a Vickers indenter impression are shown in Figure 2.

In reality, the axial width of each intermediate phase (about 1 to 3 μm) was much narrower than the indentation size. While an intermediate phase was measured, the indenter would inevitably cover the other adjacent phases, leading to an imprecise Young’s modulus of the phase. Therefore, under the premise of ignoring the interaction between two phases, according to the equilibrium condition of the force, the Young’s modulus of multiphase material is determined by [25]:

(2) $$E_{0}=\sum_{i=1}^{n}E_{i}V_{i}$$

where *E_0_* is the Young’s modulus of multiphase material (GPa), *E_i_* is the Young’s modulus of each phase (GPa), and *V_i_* is the volume fraction of each phase in multiphase material. It is noteworthy that the indenter would be pressed on a number of phases when using this method to characterize the narrow width of phases. The accurate value of *c* (the crack length) is measureable because the crack generally sprouted from the apex of the rectangular pyramid indenter and propagated along the direction of the weld interface in a single phase. Nevertheless, only comprehensive *H_v_* (the Vickers hardness) and *E* (the Young’s modulus) of multiple phases could be obtained. Consequently, this is a semi-quantitative calculation method and limited in characterizing the fracture toughness of this kind of weld interface.

**Figure 3:**
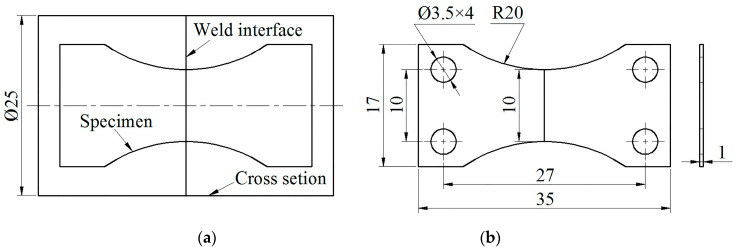
Micro-tensile specimen (mm): (**a**) sampling position and (**b**) overall dimension.

The dynamic in-situ tensile test (i.e., micro-tensile) was performed using a scanning electron microscope (SEM, Super55, Zeiss, Oberkochen, Germany) with an in-situ tensile experiment table (Microtest5000, Gatan, Pleasanton, CA, USA, 5000 N machine capacity). The tensile specimens were made from the aforementioned cross section of the joint through sampling, mechanical grinding and polishing. The sampling position and overall dimension of the micro-tensile specimens are described in Figure 3. When the tensile force was increased by 100 N with a loading speed of 0.1 mm/min, the loading was suspended. Simultaneously, the slip phenomenon, the crack initiation and crack propagation at the weld interface were detected. Then the tensile force continued to increase until the specimens were broken. Additionally, the micro-fracture position of the joint was observed and the result was compared with the macro-fracture position.

## 3. Results and Discussion

**Figure 4:**
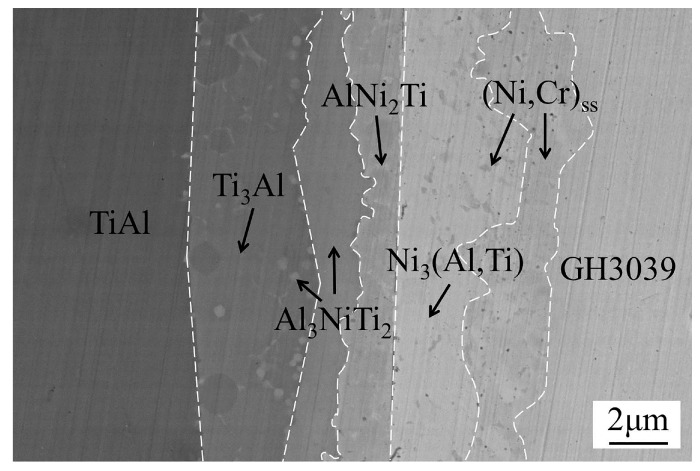
Interfacial microstructure of the friction welded joint (at the position 1 marked in Figure 1).

The intermediate phases of the friction welded joint between TiAl and GH3039 have been determined using an X-ray diffraction (XRD, X’Pert PRO, PANalytical, Almelo, Netherlands) and an electron probe microanalysis (EPMA, JXA-8100, JEOL, Tokyo, Japan) [7]. As displayed in Figure 4, the typical microstructure is TiAl/Ti_3_Al + Al_3_NiTi_2_/Al_3_NiTi_2_/AlNi_2_Ti/Ni_3_(Al,Ti) + (Ni,Cr)_ss_/(Ni,Cr)_ss_/GH3039.

### 3.1. Nanoindentation

**Figure 5:**
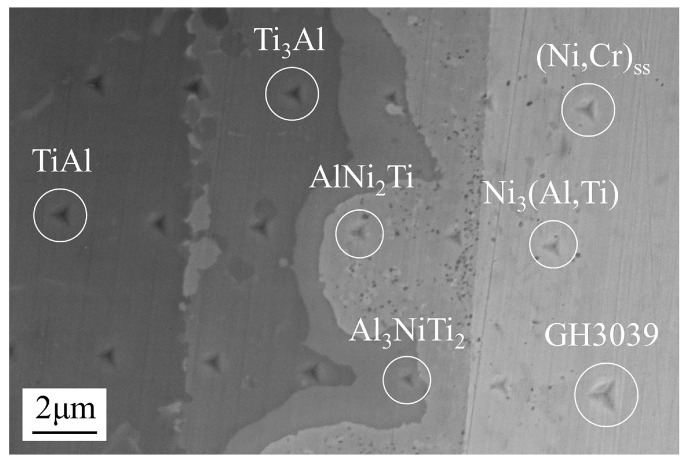
Nanoindentation impression of the friction welding interface (at the position 2 marked in Figure 1).

**Figure 6:**
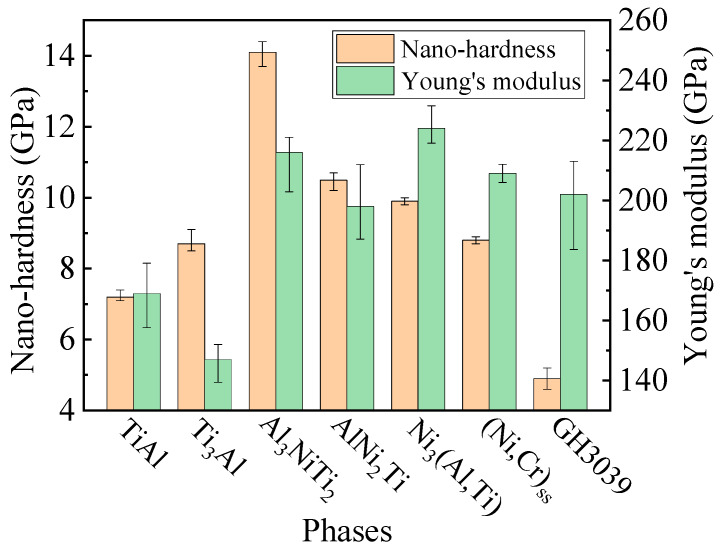
Nano-hardness and Young’s modulus of the phases.

**Figure 7:**
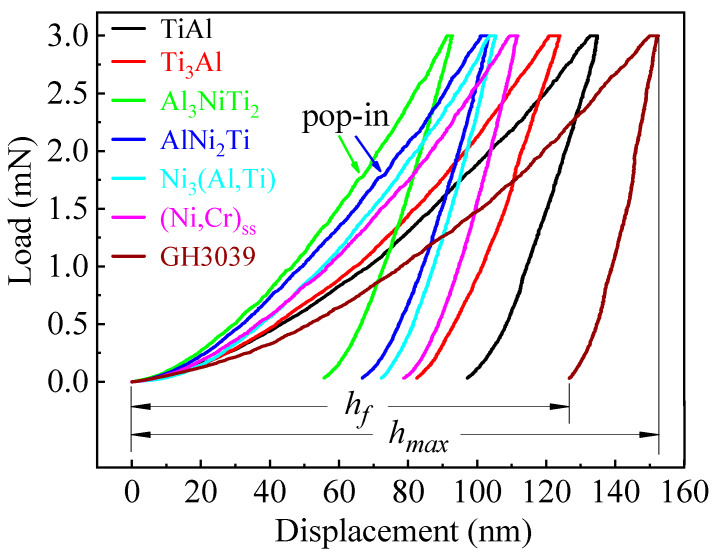
*L–D* curves of the nanoindentation.

The results of nanoindentation of the phases are shown in Figure 5, Figure 6 and Figure 7. Figure 5 visually presents the difference in hardness among phases by the size of the nanoindentation impression. It is observed that the smaller the size of the indentation impression, the higher the hardness of the phase. The indentation size of Al_3_NiTi_2_ was the smallest while that of GH3039 was the largest. As shown in Figure 6, the closer to the center of the weld zone, the higher the hardness of the phase, while the nearer to the base metals, the lower the hardness of the phase. Al_3_NiTi_2_ and AlNi_2_Ti had the highest nano-hardness of 14.1 GPa and 10.5 GPa, respectively, while TiAl and GH3039 had the lowest nano-hardness of 7.2 GPa and 4.9 GPa, respectively. The Young’s modulus of each phase was obtained together with the nano-hardness, as illustrated in Figure 6.

As illustrated in Figure 7, load–displacement (*L–D*) curves of the nanoindentation were relatively smooth. The unloading curves were neither completely coincident with loading curves nor perpendicular to the X coordinate axis, which indicates that the base metals and intermediate phases are elastoplastic materials. Under the same peak load, the higher the hardness of the phase, the shallower the maximum depth of the indentation during loading and the residual depth after elastic recovery were. The elastic recovery rate can be characterized by the ratio of indentation depths, which is defined as [26]:

(3) $$\sigma_{r}=\frac{h_{max}-h_{f}}{h_{max}}\times 100$$

where *σ_r_* is the elastic recovery rate (%), *h_max_* is the achievable maximum depth during loading (nm), and *h_f_* is the residual depth after unloading (nm). The elastic recovery rates of the two hardest phases, Al_3_NiTi_2_ and AlNi_2_Ti, were 39.9% and 35.5%, respectively, while those of the two softest phases, TiAl and GH3039, were 27.9% and 16.8%, respectively. It is concluded that the harder phases such as Al_3_NiTi_2_ and AlNi_2_Ti generated more elastic deformation, while the softer phases such as TiAl and GH3039 generally produced less elastic deformation in the nanoindentation test.

The displacement mutation in the depth data of the *L–D* curves, the so-called pop-in, is initiated by the nucleation and multiplication of new dislocations [10]. That is, pop-in can be ascribed to the dislocation nucleation and subsequent dislocation multiplication. As displayed in Figure 7, the obvious pop-in event only existed in the *L–D* curves of Al_3_NiTi_2_ and AlNi_2_Ti at the same peak load. The nucleation of dislocations represented by the pop-in occurs when the maximum shear stress underneath the indenter approaches the permanent deformation strength of the intermediate phases [27]. Hence, the pop-in phenomenon demonstrates that smaller external force (stress) is required for the dislocation nucleation and subsequent dislocation multiplication in the two harder phases (Al_3_NiTi_2_ and AlNi_2_Ti) when compared with the other five phases.

### 3.2. Fracture Toughness

It is observed from the aforementioned results that the hardness of Al–Ni–Ti ternary brittle intermetallic compounds, Al_3_NiTi_2_ and AlNi_2_Ti, was the highest. However, high hardness of intermediate phases with layered distribution may weaken the welded joints [23,28]. The analysis of the fracture surfaces and the tendency of cracks propagation also indicated that the cracks were most inclined to be generated in the Al_3_NiTi_2_ and AlNi_2_Ti phases when the joint was subjected to tensile force [7]. Therefore, the fracture toughness of the intermediate phases was surveyed by the indentation technique.

**Figure 8:**
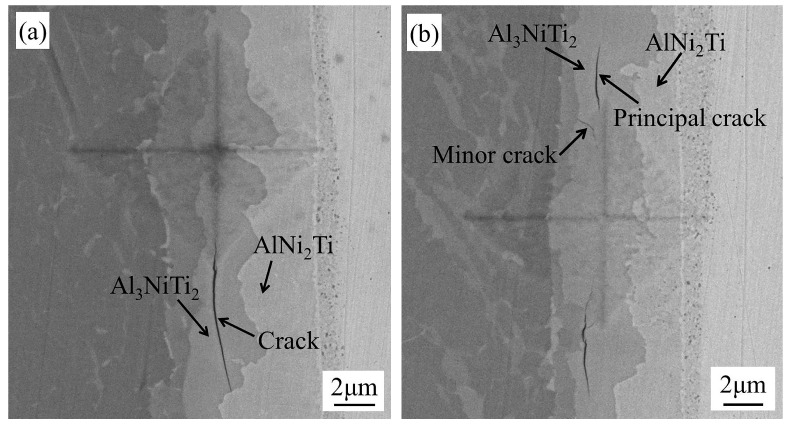

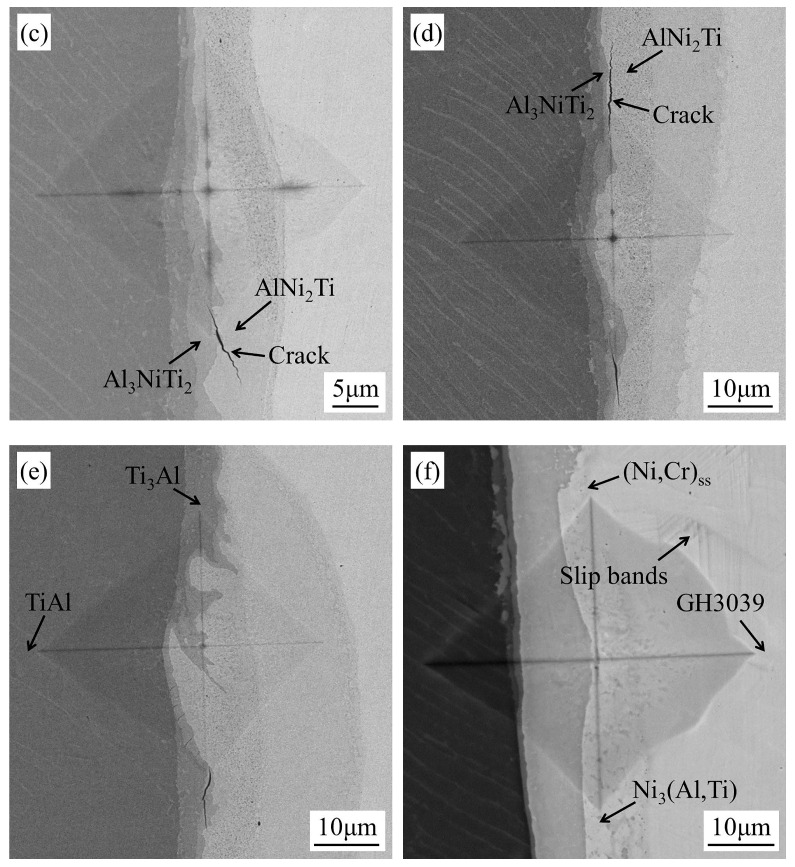
Morphology of the indentation cracks under peak loads of: (**a**), (**b**) 0.5 N; (**c**) 3 N; and (**d**), (**e**), (**f**) 5 N (at the position 4 marked in Figure 1).

Figure 8 presents the morphology of the indentation cracks under diverse peak loads. As seen in Figure 8a,b, the cracks originated from Al_3_NiTi_2_ first when the peak load increased from 0.1 to 0.5 N. As shown in Figure 8a, the crack sprouted from the cross apex of the indenter and propagated further along the direction of the weld interface in Al_3_NiTi_2_. The crack terminated at the phase boundary of Al_3_NiTi_2_ and AlNi_2_Ti, which indicates that the fracture toughness of AlNi_2_Ti was superior to that of Al_3_NiTi_2_. It can also be found in Figure 8b that in addition to the two principal cracks generated around the cross tip of the indenter, a minor crack was also produced at the edge of the rectangular pyramid. Referring to (1), (2) and the Young’s modulus of each phase (shown in Figure 6), the average fracture toughness of Al_3_NiTi_2_ was determined to be 1.7 MPa·m^1/2^. When the peak load increased to 3 N, as illustrated in Figure 8c, the crack sprouted from Al_3_NiTi_2_ and propagated to AlNi_2_Ti, i.e., the crack began to be produced in AlNi_2_Ti. Figure 8d reveals that an unbroken crack was generated and extended in AlNi_2_Ti under the peak load of 5 N, and the average fracture toughness of AlNi_2_Ti was determined to be 2.7 MPa·m^1/2^. Eventually, when the peak load increased to at most 10 N, as displayed in Figure 8e,f, no cracks were formed in TiAl, Ti_3_Al, Ni_3_(Al,Ti), (Ni,Cr)_ss_ and GH3039. Consequently, the fracture toughness of these phases could not be determined. A large number of slip bands were generated in GH3039 due to its severe plastic deformation, which led to the distorted indentation morphology. In summary, the fracture toughness of Al_3_NiTi_2_ and AlNi_2_Ti was lower than that of the other phases.

### 3.3. In-Situ Tensile

**Figure 9:**
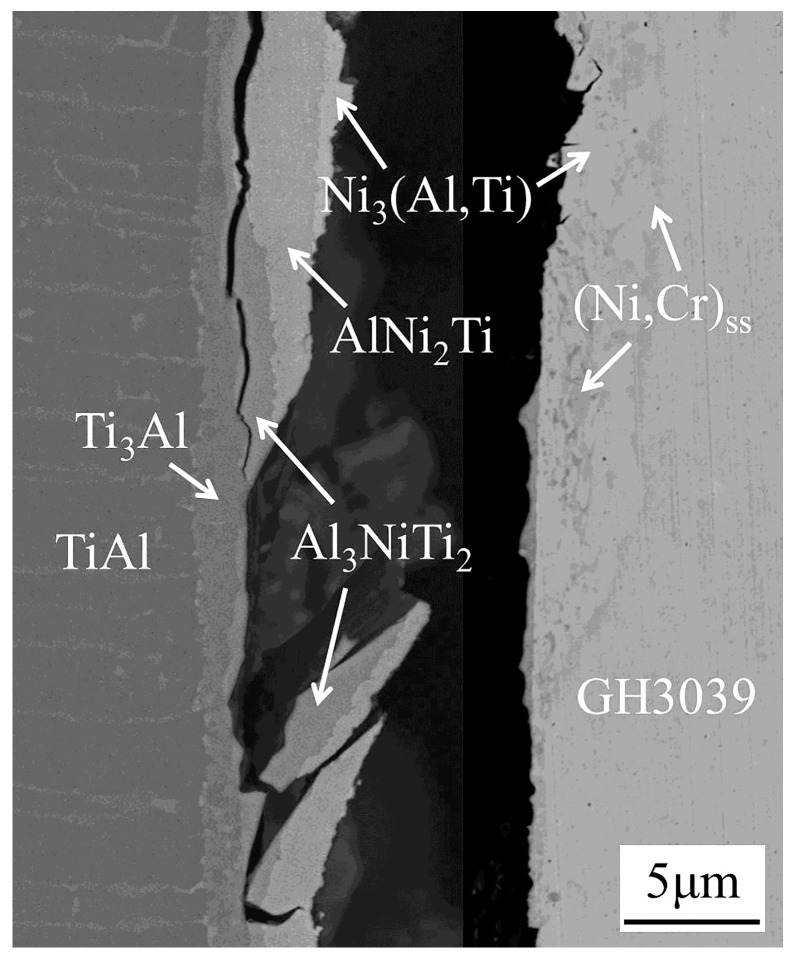
Interfacial microstructure after the in-situ tensile test in SEM (at the position 3 marked in Figure 1)**.**

The intermediate phases with low fracture toughness would inevitably deteriorate the joint strength. Hence, it is important to determine the fracture position at the weld interface by the in-situ tensile test. When the tensile force increased from 0 to 1200 N, neither slip nor crack was observed. When the tensile force increased to 1270 N, the fracture of the specimen happened instantaneously. That is, the micro-tensile strength of the joint was 127 MPa. Due to the layered distribution of brittle phases at the interface, the cracks propagated rapidly once they were generated. Consequently, the initiation and dynamic propagation process of the cracks were not observed. The advantage of the in-situ tensile test is that the distribution relationship of each intermediate phase at the interface after fracture can be observed on the fly. However, the fracture position of the joint after the conventional tensile cannot be directly observed because of the narrow width of the phases. Figure 9 shows the interfacial microstructure after the in-situ tensile test in SEM. The fracture mainly happened at the phase layers of Al_3_NiTi_2_ and AlNi_2_Ti. The cracks sprouted from Al_3_NiTi_2_ and AlNi_2_Ti and then promptly spread throughout the entire intermediate phase zone. The result shown in Figure 9 was consistent with that from the early macroscopic tensile test [7].

The fracture occurred at the phase layers of Al_3_NiTi_2_ and AlNi_2_Ti, which could be explained by the dislocation nucleation and the fracture toughness. As derived from Section 3.1, less stress is required for the dislocation nucleation and subsequent dislocation multiplication in Al_3_NiTi_2_ and AlNi_2_Ti when compared with the other five phases. That is, under the same tensile stress, the dislocation nucleation first occurs in Al_3_NiTi_2_ and AlNi_2_Ti and the number and density of dislocations would gradually increase via multiplication. As a result, dislocations are finally aggregated to form plane and volume defects which lead to the production of cracks in Al_3_NiTi_2_ and AlNi_2_Ti. Because of the lower fracture toughness of Al_3_NiTi_2_ and AlNi_2_Ti, the critical stress required for the unstable propagation of the cracks is smaller. Once cracks occur, they would first propagate in the two phases and result in the fracture at the joint. Therefore, the maximum tensile stress that Al_3_NiTi_2_ and AlNi_2_Ti can withstand is the lowest among seven phases at the weld interface. Al_3_NiTi_2_ and AlNi_2_Ti are the two weakest phases at the interface and critically deteriorate the TiAl/GH3039 friction welded joints.

## 4. Conclusions

The micromechanical properties of the friction welded joint between TiAl and GH3039 were characterized by nano-hardness, fracture toughness and in-situ tensile. The main conclusions are as follows:

(1) Al_3_NiTi_2_ and AlNi_2_Ti had the highest nano-hardness and elastic recovery rates, while that of TiAl and GH3039 base metals were the lowest. The harder the materials, the more prone they are to elastic deformation;

(2) The fracture toughness of Al_3_NiTi_2_ and AlNi_2_Ti with higher elasticity reached 1.7 MPa·m^1/2^ and 2.7 MPa·m^1/2^, respectively, which were lower than that of the other phases;

(3) The fracture occurring at the brittle phase layers of Al_3_NiTi_2_ and AlNi_2_Ti was observed by the in-situ tensile test. Al_3_NiTi_2_ and AlNi_2_Ti are the weakest phases at the interface and their poor fracture toughness is the primary cause of deterioration of the joint strength;

(4) In the future, attention should be paid to the establishment of the relationship among the process parameters, the tensile strength and the thickness of brittle intermediate phase layers. The joint performance would be ultimately promoted through controlling the thickness of brittle phase layers.

## Figures and Tables

**Table 1:** Chemical compositions of the base metals.

Materials	Chemical Composition (at.%)							
	Ti	Al	Ni	Cr	V	Fe	Mo	Nb
TiAl	49.0	47.5	−	1.0	2.5	−	−	−
GH3039	0.6	1.1	69.6	22.6	−	2.9	1.2	0.7

## References

[1] Du Z., Zhang K., Lu Z., Jiang S. (2018). Microstructure and mechanical properties of vacuum diffusion bonding joints for γ-TiAl based alloy. Vacuum.

[2] Song Y., Dou Z., Zhang T., Liu Y. (2019). A novel continuous and controllable method for fabrication of as-cast TiAl alloy. J. Alloy. Compd..

[3] Hauschildt K., Stark A., Schell N., Müller M., Pyczak F. (2019). The transient liquid phase bonding process of a γ-TiAl alloy with brazing solders containing Fe or Ni. Intermetallics.

[4] Cai X., Sun D., Li H., Guo H., Gu X., Zhao Z. (2017). Microstructure characteristics and mechanical properties of laser-welded joint of γ-TiAl alloy with pure Ti filler metal. Opt. Laser Technol..

[5] Dong H., Yang Z., Wang Z., Deng D., Dong C. (2015). CuTiNiZrV Amorphous Alloy Foils for Vacuum Brazing of TiAl Alloy to 40Cr Steel. J. Mater. Sci. Technol..

[6] Li Y., He P., Feng J. (2006). Interface structure and mechanical properties of the TiAl/42CrMo steel joint vacuum brazed with Ag-Cu/Ti/Ag-Cu filler metal. Scr. Mater..

[7] Du S., Wang S., Ding K. (2020). A novel method of friction-diffusion welding between TiAl alloy and GH3039 high temperature alloy. J. Manuf. Process..

[8] Iracheta O., Bennett C.J., Sun W. (2016). Characterization of material property variation across an inertia friction welded CrMoV steel component using the inverse analysis of nanoindentation data. Int. J. Mech. Sci..

[9] Hsueh C., Liao M., Wang S., Tsai Y., Yang J., Wu R., Lee W. (2018). Size effect and strain induced double twin by nanoindentation in DSS weld metal of vibration-assisted GTAW. Mater. Chem. Phys..

[10] Liu W., Wang Y., Ma Y., Yu Q., Huang Y. (2016). Nanoindentation study on micromechanical behaviors of Au–Ni–Sn intermetallic layers in Au–20Sn/Ni solder joints. Mater. Sci. Eng. A.

[11] Cabibbo M., Forcellese A., Mehtedi M.E., Simoncini M. (2014). Double side friction stir welding of AA6082 sheets: Microstructure and nanoindentation characterization. Mater. Sci. Eng. A.

[12] Chu Q., Li J., Tong X., Xu S., Zhang M., Yan C. (2019). Nanoindentation and microstructure analysis of Ti/Fe dissimilar joint. Mater. Lett..

[13] Yu W., Fan M., Shi J., Xue F., Chen X., Liu H. (2018). A comparison between fracture toughness at different locations of SMAW and GTAW welded joints of primary coolant piping. Eng. Fract. Mech..

[14] Li X., Li K., Li S., Wu Y., Cai Z., Pan J. (2020). Microstructure and high temperature fracture toughness of NG-TIG welded Inconel 617B superalloy. J. Mater. Sci. Technol..

[15] Sebastiani M., Johanns K.E., Herbert E.G., Pharr G.M. (2015). Measurement of fracture toughness by nanoindentation methods: Recent advances and future challenges. Curr. Opin. Solid State Mater. Sci..

[16] Munther M., Palma T., Tavangarian F., Beheshti A., Davami K. (2020). Nanomechanical properties of additively and traditionally manufactured nickel-chromium-based superalloys through instrumented nanoindentation. Manuf. Lett..

[17] Cai X., Xu Y., Zhong L., Liu M. (2017). Fracture toughness of WC-Fe cermet in W-WC-Fe composite by nanoindentation. J. Alloys Compd..

[18] Casellas D., Caro J., Molas S., Prado J., Valls I. (2007). Fracture toughness of carbides in tool steels evaluated by nanoindentation. Acta Mater..

[19] Chu Q., Zhang M., Li J., Yan F., Yan C. (2018). Investigation of microstructure and fracture toughness of Fe-Zr welded joints. Mater. Lett..

[20] Ahn J., He E., Chen L., Dear J., Shao Z., Davies C. (2018). In-situ micro-tensile testing of AA2024-T3 fibre laser welds with digital image correlation as a function of welding speed. Int. J. Light. Mater. Manuf..

[21] Yang B., Xuan F., Chen J. (2018). Evaluation of the microstructure related strength of CrMoV weldment by using the in-situ tensile test of miniature specimen. Mater. Sci. Eng. A.

[22] Zhang K., Ni L., Lei Z., Chen Y., Hu X. (2017). In situ investigation of the tensile deformation of laser welded Ti_2_AlNb joints. Mater. Charact..

[23] Li Y., Lv M., Feng J., He P. (2014). Characteristics of reaction phases and effects of phases on mechanical properties of TiAl/42CrMo steel brazed joint. Trans. China Weld. Inst..

[24] Anstis G.R., Chantikul P., Lawn B.R., Marshall D.B. (1981). A Critical Evaluation of Indentation Techniques for Measuring Fracture Toughness: I, Direct Crack Measurements. J. Am. Ceram. Soc..

[25] Ji S. (2004). Generalized means as an approach for predicting Young’s moduli of multiphase materials. Mater. Sci. Eng. A.

[26] Fang X., Li C., Sun L., Sun H., Jiang Z. (2020). Hardness and friction coefficient of fused silica under scratching considering elastic recovery. Ceram. Int..

[27] Shim S., Bei H., George E.P., Pharr G.M. (2008). A different type of indentation size effect. Scr. Mater..

[28] Tetsui T. (2001). Effects of brazing filler on properties of brazed joints between TiAl and metallic materials. Intermetallics.

